# Cardiac remodelling in a swine model of chronic thromboembolic pulmonary hypertension: comparison of right *vs*. left ventricle

**DOI:** 10.1113/JP277896

**Published:** 2019-07-25

**Authors:** Kelly Stam, Zongye Cai, Nikki van der Velde, Richard van Duin, Esther Lam, Jolanda van der Velden, Alexander Hirsch, Dirk J Duncker, Daphne Merkus

**Affiliations:** ^1^ Department of Cardiology, Erasmus MC University Medical Center Rotterdam Rotterdam The Netherlands; ^2^ Department of Radiology and Nuclear Medicine, Erasmus MC University Medical Center Rotterdam Rotterdam The Netherlands; ^3^ Amsterdam UMC Vrije Universiteit Amsterdam, Physiology, Amsterdam Cardiovascular Sciences Amsterdam The Netherlands

**Keywords:** Pulmonary hypertension, Exercise, Right ventricular hypertrophy

## Abstract

**Key points:**

- Right ventricle (RV) function is the most important determinant of survival and quality of life in patients with chronic thromboembolic pulmonary hypertension (CTEPH).
- The changes in right and left ventricle gene expression that contribute to ventricular remodelling are incompletely investigated.
- RV remodelling in our CTEPH swine model is associated with increased expression of the genes involved in inflammation (TGFβ), oxidative stress (ROCK2, NOX1 and NOX4), and apoptosis (BCL2 and caspase‐3).
- Alterations in ROCK2 expression correlated inversely with RV contractile reserve during exercise.
- Since ROCK2 has been shown to be involved in hypertrophy, oxidative stress, fibrosis and endothelial dysfunction, ROCK2 inhibition may present a viable therapeutic target in CTEPH.

**Abstract:**

Right ventricle (RV) function is the most important determinant of survival and quality of life in patients with chronic thromboembolic pulmonary hypertension (CTEPH). The present study investigated whether the increased cardiac afterload is associated with (i) cardiac remodelling and hypertrophic signalling; (ii) changes in angiogenic factors and capillary density; and (iii) inflammatory changes associated with oxidative stress and interstitial fibrosis. CTEPH was induced in eight chronically instrumented swine by chronic nitric oxide synthase inhibition and up to five weekly pulmonary embolizations. Nine healthy swine served as a control. After 9 weeks, RV function was assessed by single beat analysis of RV–pulmonary artery (PA) coupling at rest and during exercise, as well as by cardiac magnetic resonance imaging. Subsequently, the heart was excised and RV and left ventricle (LV) tissues were processed for molecular and histological analyses. Swine with CTEPH exhibited significant RV hypertrophy in response to the elevated PA pressure. RV–PA coupling was significantly reduced, correlated inversely with pulmonary vascular resistance and did not increase during exercise in CTEPH swine. Expression of genes associated with hypertrophy (BNP), inflammation (TGFβ), oxidative stress (ROCK2, NOX1 and NOX4), apoptosis (BCL2 and caspase‐3) and angiogenesis (VEGFA) were increased in the RV of CTEPH swine and correlated inversely with RV–PA coupling during exercise. In the LV, only significant changes in ROCK2 gene‐expression occurred. In conclusion, RV remodelling in our CTEPH swine model is associated with increased expression of genes involved in inflammation and oxidative stress, suggesting that these processes contribute to RV remodelling and dysfunction in CTEPH and hence represent potential therapeutic targets.

## Key points

- Right ventricle (RV) function is the most important determinant of survival and quality of life in patients with chronic thromboembolic pulmonary hypertension (CTEPH).
- The changes in right and left ventricle gene expression that contribute to ventricular remodelling are incompletely investigated.
- RV remodelling in our CTEPH swine model is associated with increased expression of the genes involved in inflammation (TGFβ), oxidative stress (ROCK2, NOX1 and NOX4), and apoptosis (BCL2 and caspase‐3).
- Alterations in ROCK2 expression correlated inversely with RV contractile reserve during exercise.
- Since ROCK2 has been shown to be involved in hypertrophy, oxidative stress, fibrosis and endothelial dysfunction, ROCK2 inhibition may present a viable therapeutic target in CTEPH.

## Introduction

Chronic thromboembolic pulmonary hypertension (CTEPH) develops in a subset of patients after acute pulmonary embolism (Lang *et al*. 2016; Simonneau *et al*. 2017). In CTEPH, pulmonary vascular resistance, which is initially elevated because of obstructions in the larger pulmonary arteries, is further increased by pulmonary microvascular remodelling (Lang *et al*. 2016; Simonneau *et al*. 2017). This increased pulmonary vascular resistance augments afterload of the right ventricle (RV), thereby resulting in RV dilatation and RV hypertrophy. RV structural and functional adaptability are important determinants of functional capacity and survival in patients with CTEPH (Hardziyenka *et al*. 2011; van de Veerdonk *et al*. 2016; Claeys *et al*. 2019). Thus, RV–pulmonary arterial uncoupling is associated with reduced exercise capacity (Claeys *et al*. 2019) and patients with RV dilatation have a worse prognosis compared to patients with preserved RV function and geometry (Blumberg *et al*. 2013; Grosse *et al*. 2018). Furthermore, it is increasingly recognized that RV dysfunction may also influence the left ventricle (LV), both mechanically, via direct mechanical interaction and changes in LV filling, by inducing interventricular asynchrony (Marcus *et al*. 2008; Vonk Noordegraaf *et al*. 2017), as well as via activation of inflammatory pathways, which may be the result of low grade systemic inflammation in combination with neurohumoral activation because of reduced cardiac output (Dell'Italia, 2011; Hardziyenka *et al*. 2011; Naeije & Badagliacca, 2017).

In CTEPH, pulmonary obstructive lesions can be located both proximally and distally. Distal pulmonary lesions have recently been shown to be associated with worse prognosis, in part because distal pulmonary lesions are currently considered inoperable, and in part because distal pulmonary emboli are associated with worse RV function (Grosse *et al*. 2018). Furthermore, also in patients with chronic thromboembolic disease, even without overt pulmonary hypertension (PH), RV dysfunction has been observed (McCabe *et al*. 2014), which is associated with an impaired exercise capacity (Claeys *et al*. 2019).

The factor(s) that predispose(s) to RV failure currently remain unknown. Unlike pulmonary arterial hypertension, which is usually only detected in a very advanced stage of the disease, CTEPH occurs mostly after acute pulmonary embolism, which, despite the fact that this first pulmonary embolism may also go unnoticed (Ende‐Verhaar *et al*, 2017), may allow earlier intervention in the process of RV remodelling and adaptation. Mild RV dysfunction is characterized by a deterioration of RV diastolic function (i.e. relaxation), whereas RV contraction is still preserved (McCabe *et al*. 2014). The main determinant of cardiac diastolic function is cardiac stiffness, which is negatively influenced by interstitial fibrosis, as well as by changes in isoform expression of the ‘cardiac spring protein’ titin (Rain *et al*. 2016). Furthermore, it has been proposed that the failure of angiogenesis to keep up with RV hypertrophy, which results in reduced capillary densities and concommittant RV perfusion abnormalities, is a key determinant that discriminates between adaptive RV hypertrophy and RV failure (Frump *et al*. 2018).

We have recently developed a swine model, in which a combination of endothelial dysfunction by nitric oxide synthase (NOS) inhibition with pulmonary embolizations with microspheres of ∼700 µm in diameter resulted in the development of CTEPH with distal pulmonary microvascular remodelling (Stam *et al*. 2018a,*b*). In CTEPH animals, the increased afterload was accompanied by RV hypertrophy, which resulted in preservation of RV function at rest, although stroke volume (SV) decreased with increasing exercise intensity, suggesting mild RV dysfunction (Stam *et al*. 2018a).

In the present study, we investigated the changes in RV and LV geometry and morphology in CTEPH, as well as the concomittant changes in gene expression that may contribute to these changes. Specifically, we investigated whether the increased RV afterload is associated with (i) cardiac remodelling and hypertrophic signalling; (ii) changes in angiogenic factors and capillary density; and (iii) inflammatory changes associated with oxidative stress and interstitial fibrosis.

## Methods

### Ethical approval

Animal studies were performed following the ‘Guiding Principles for the Care and Use of Laboratory Animals’ as approved by the Council of the American Physiological Society, and with approval of the Animal Care Committee of the Erasmus University Medical Center (EMC3158, 109‐13‐09). The authors understand the ethical principles under which *The Journal of Physiology* operates and hereby declare that this work complies with the journal's animal ethics checklist (Grundy, 2015). Twenty‐three crossbred Landrace × Yorkshire swine of either sex obtained from a commercial breeder (3 months old, weighing 22 ± 1 kg) entered the study. Swine were individually housed in the animal facility of the Erasmus University Medical Center, fed twice a day and had free access to drinking water. Our experimental protocol consists of a chronic instrumentation, followed by induction of CTEPH in 12 animals via a combination of NOS‐inhibition with l‐*N* ^ω^‐nitroarginine methyl ester (l‐NAME) and up to five weekly repeated embolizations with microspheres (see below for details). Mortality as a result of acute cardiopulmonary failure upon CTEPH induction occurred in two animals. Two animals were excluded because of catheter failure (one control, one CTEPH), whereas two animals (one control, one CTEPH) had to be killed following repeated infections because of the catheters and were not included. Only animals that completed the protocol are included in the reported numbers (*n*).

### Chronic instrumentation

Animals were chronically instrumented as described previously (De Wijs‐Meijler *et al*. 2016; Stam *et al*. 2018a). In short, after an overnight fast, swine were sedated with an i.m. injection of tiletamine/zolazepam (5 mg kg^−1^), xylazine (2.25 mg kg^−1^) and atropine (1 mg), intubated and ventilated with a mixture of O_2_ and N_2_ (1:2 v/v) to which 2% (v/v) isoflurane was added to maintain anaesthesia. Under sterile conditions, a left thoracotomy in the fourth intercostal space was performed, the pericardium was opened and fluid‐filled polyvinylchloride catheters (Braun Medical Inc., Bethlehem, PA, USA) were placed in the RV, pulmonary artery and aorta for blood pressure measurement. A flow probe (Transonic Systems Inc., Ithaca, NY, USA) was positioned around the ascending aorta for measurement of cardiac output (CO). The catheters were tunnelled to the back, the chest was closed, and animals were allowed to recover for 1 week, receiving analgesia (0.015 mg kg^−1^ buprenorphine i.m. and a slow‐release fentanyl patch 12 µg h^−1^ for 48 h) on the day of the surgery and daily i.v. antibiotic prophylaxis (25 mg kg^−1^ amoxicillin) for 7 days (De Wijs‐Meijler *et al*. 2016).

### CTEPH induction

Following the recovery week, CTEPH was successfully induced in eight animals (four males, four females) as described previously (Stam *et al*. 2018a,*b*). In short, on the first day, the animals were given the NOS‐inhibitor l‐NAME (10 mg kg^−1^ i.v.; Enzo Life Sciences International Inc, NY, USA) as a bolus infusion. On subsequent days, the dose of l‐NAME was increased by 10 mg kg^−1^ per day up to 30 mg kg^−1^ i.v., which was maintained until 1 week before the end of the study (Rees *et al*. 1990; Matsunaga *et al*. 2000). l‐NAME exhibits *K*_i_ values of 15 nm, 39 nm and 4.4 µm for neuronal NOS (bovine), endothelial nitric oxide synthase (eNOS) (human) and inducible NOS (mouse) (Buckner *et al*. 1988; Furfine *et al*. 1993; Garvey *et al*. 1994). Four days after the beginning of l‐NAME administration, microsphere infusions were started. Polyethylene microspheres (diameter 600–710 µm, density 1.13 g cm^−3^, 500 mg, corresponding to ∼2500 microspheres; Cospheric LLC, Santa Barbara, California, USA) were suspended in 50 mL of autologous blood with 2500 IU. heparin and slowly infused into the RV while monitoring mean pulmonary artery pressure (mPAP). Microsphere infusions were repeated until the mPAP reached ∼60 mmHg, or the arterial $P_{aO_{2}}$ dropped below ∼40 mmHg, as measured at rest 30 min after infusion, or when a maximum of 3 g (∼15000) microspheres was infused on 1 day based on the assumption that the porcine lungs contain ∼25000 small arteries of this diameter. In the subsequent 4 weeks, haemodynamics were assessed weekly, and microsphere infusions were repeated when mPAP was <25 mmHg and $P_{aO_{2}}$ >70 mmHg, as described above. During the final 5 weeks of follow‐up, no microsphere infusions were performed, whereas l‐NAME administration was discontinued 1 week before death (Stam *et al*. 2018a,*b*).

Seven sham‐operated animals (three males, four females), which did not receive l‐NAME or microspheres, and two additional healthy animals (two females), which were not operated on, served as controls.

### *In vivo* experiments

#### Haemodynamic studies

Haemodynamic studies were performed 10 weeks after surgery. With swine standing quietly on a motor‐driven treadmill and during exercise at 4 km h^−1^, CO, PAP, aorta pressure (AoP) and RV pressure were recorded continuously (Duncker *et al*. 1998; De Wijs‐Meijler *et al*. 2016). Two animals were unable to perform exercise testing.

**Figure 1:**
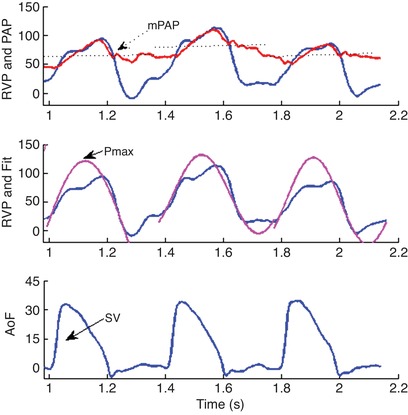
Typical example of RV–PA coupling analysis showing haemodynamic signals (three beats) and their derivatives *P*_max_ was determined as the maximal value from a sine‐fit of RV pressure (RVP), *P*_es_ was estimated to equal mPAP, whereas SV was calculated as the time‐integral of aorta flow (AoF).

Digital recording and offline analysis of haemodynamic data were performed as described previously (Duncker *et al*. 1998; Stubenitsky *et al*. 1998). To account for differences in growth between animals, CO was corrected for body weight, yielding cardiac index (CI). Stroke volume index (SVi) was calculated as CI/heart rate. Total pulmonary vascular resistance index (tPVRi) and systemic vascular resistance index (SVRi) were calculated as mPAP/CI and mAoP/CI, respectively. RV function was measured by single beat analysis of RV–pulmonary artery (PA) coupling as described previously (Brimioulle *et al*. 2003), using the median value of at least 10 consecutive beats, assuming that end‐systolic PAP equals mPAP (Chemla *et al*. 1996; Brimioulle *et al*. 2003). For calculation of *E*_es_, a sine wave was fitted to the isovolumetric contraction and relaxation phases of RV contraction. The top of the sine wave has previously been shown to be a good approximation of *P*_max_, derived from isovolumetric contraction (Fig. 1). *E*_es_ was subsequently calculated as (*P*_max_ – mPAP)/SVi. *E*_a_ was calculated as mPAP/SVi. RV–PA coupling was assessed as the ratio of *E*_es_ and *E*_a_.

#### Cardiovascular magnetic resonance imaging

After completion of the haemodynamic experiments, a cardiovascular magnetic resonance (CMR) examination was performed on a 1.5 T clinical scanner with a dedicated 32‐channel phased‐array cardiac surface coil (Discovery MR450; GE Healthcare, Milwaukee, WI, US, USA) in five control (two male, three female) and six CTEPH (four male, two female) animals. For this purpose, animals were sedated and intubated as described above. During imaging, anaesthesia was maintained with pentobarbital sodium (6–12 mg kg^−1^ h^−1^ i.v.). Mechanical ventilation and breath‐holds were performed using a mobile ventilator (Carina™; Dräger Medical, Best, The Netherlands). When necessary, and always in absence of pain reflexes, muscle relaxation was temporarily achieved using pancuronium bromide (2–4 mg i.v. bolus). The imaging protocol consisted of retrospectively ECG‐gated balanced Steady‐State Free Precession cine imaging with breath‐holding (FIESTA; GE Medical Systems, Waukesha, WI, USA). Standard long‐axis and short‐axis images with full LV and RV coverage were obtained. Typical scan parameters were slice thickness 6.0 mm, slice gap 0 mm, TR/TE 3.4/1.4 ms, flip angle 75°, field of view 320 × 240 mm, acquired matrix 180 × 128 and reconstructed matrix 256 × 256. To assess dimensions, function and mass of both ventricles, LV and RV epi‐ and endocardial contours were drawn manually on end‐diastolic and end‐systolic short axis cine images. Volumes and masses were measured, and stroke volumes and ejection fractions (EF) were calculated. All volumes were indexed for body weight. QMassMR analytical software, version 8.1 (Medis BV, Leiden, The Netherlands) was used for analysis.

### Euthanasia

After completion of the *in vivo* experiments, with animals intubated and under deep anaesthesia (pentobarbital sodium, 6–12 mg kg^−1^ h^−1^ i.v.), a sternotomy was performed, ventricular fibrillation was induced using a 9 V battery and the heart was immediately excised. To assess relative RV hypertrophy, the heart was sectioned into RV free wall and LV (including septum) and then weighed. RV hypertrophy was assessed using the Fulton index (RV/LV). Parts of the LV anterior wall and RV were snap frozen in liquid nitrogen within 10 min after excision for molecular analyses and fixated in formaldehyde for histological analysis.

### Histology

LV and RV tissues were fixated in 3.5–4% buffered formaldehyde for a minimum of 24 h and embedded in paraffin wax. Subsequently, 5 µm sections were cut and stained with (i) Gomori to assess cardiomyocyte cross‐section area (CSA); (ii) lectin to assess capillary density; and (iii) Picrosirius red to assess collagen content (Sorop *et al*. 2018). The stained sections were scanned using a Hamamatsu NDP scanner (Hamamatsu Nanozoomer 2.0 HT; Hamamatsu Photonics KK, Hamamatsu City, Japan). Morphometric measurements of CSA and capillary density (expressed as number of capillaries per mm^2^ and per cardiomyocyte) were performed using Clemex Vision Professional Edition (Clemex Technologies Inc., Corporate Headquarters, Quebec, Canada), whereas collagen content was analysed using BioPixiQ (BioPix AB, Gothenburg, Sweden) as described previously (Sorop *et al*. 2018).

### Real‐time quantitative PCR

Total RNA was extracted from snap frozen LV and RV tissues with a RNeasy Fibrous Tissue Mini Kit (Qiagen, Venlo, The Netherlands) as described previously (Stam *et al*. 2018a). RNA integrity was confirmed using a Bioanalyzer (2100 Bioanalyzer; Agilent, Santa Clara, CA, USA). cDNA was synthesized from 500 ng of total RNA with a SensiFAST cDNA Synthesis Kit (Bioline, London, UK). A quantitative RT‐PCR (CFX‐96; Bio‐Rad, Hercules, California, USA) was performed with a SensiFAST SYBR & Fluorescein Kit (Bioline). Target genes mRNA expression levels were normalized against β‐actin, glyceraldehyde‐3‐phosphate dehydrogenase and cyclophilin using the ΔΔ*C*_t_ method with the gene study function in CFX manager software (Bio‐Rad). All primer sequences are presented in Table 1.

**Table 1:** Primer sequences used for the quantitative PCR

	Sequence	
Genes	Forward	Reverse
β‐actin	TCCCTGGAGAAGAGCTACGA	AGCACCGTGTTGGCGTAGAG
Cyclophilin	AGACAGCAGAAAACTTCCGTG	AAGATGCCAGGACCCGTATG
GAPDH	GCTCATTTCCTCGTACGAC	GAGGGCCTCTCTCCTC
α‐SMA	GGACCCTGTGAAGCACCAG	GGGCAACACGAAGCTCATTG
β‐MHC	AGATGAACGAGCATCGGAGC	TACTGTTCCCGAAGCAGGTCAG
ANP	TGAACCCAGCCCAGAGAGAT	CAGTCCACTCTGTGCTCCAA
BNP	CAAGTCCTCCGGGGAATACG	TACCTCCTGAGCACATTGCAG
SERCA2a	GACAATGGCGCTGTCTGTTC	ATCGGTACATGCCGAGAACG
PLN	TTCCAGCTAAACACCGATAAGA	AGGCAGCCTTGGCTGTTTAT
BCL2	GATAACGGAGGCTGGGATGC	TTATGGCCCAGATAGGCACC
BCLXL	TGAGTCGGATCGCAACTTGG	GCTAGAGTCATGCCCGTCAG
Casp3	GCTGCAAATCTCAGGGAGAC	CATGGCTTAGAAGCACGCAA
eNOS	GGACACACGGCTAGAAGAGC	TCCGTTTGGGGCTGAAGATG
VEGFA	ACTGAGGAGTTCAACATCGCC	CATTTACACGTCTGCGGATCTT
HIF1α	TTTACTCATCCGTGCGACCA	AGCTCCGCTGTGTATTTTGC
HIF2α	GTCGAAGATCAGCACACGGA	CACCGCTCCTGAGACTCTTC
IL‐6	CTCCAGAAAGAGTATGAGAGC	AGCAGGCCGGCATTTGTGGTG
TNF‐α	TGCACTTCGAGGTTATCGGCC	CCCACTCTGCCATTGGAGCTG
IFN‐γ	GAAGAATTGGAAAGAGGAGAGTGAC	TGCTCCTTTGAATGGCCTGG
TGF‐β1	GTGGAAAGCGGCAACCAAAT	CACTGAGGCGAAAACCCTCT
BMPR2	GGATGCTGACAGGAGATCGT	CTGGCGGTTTGCAAAGGAAA
PAI‐1	TGAATGAGAGCGGCACGGTG	TTGTGCCGCACCACGAACAG
Id‐1	GGAGTTGGAGCTGAACTCGG	GCGATCGTCCGCTGGAACAC
NOX1	CCATTCATATTCGAGCAGCAGG	AACATCCTCACTGACAGTGCC
NOX2	TTGGCGATCTCAGCAGAAGG	GAGGTCAGGGTGAAAGGGTG
NOX4	GCAGACTTACTCTGTGTGTTG	CCATCTGTCTGACTGAGGTAC
PCNA	GAACCTCACCAGCATGTCCAA	TAGTGCCAAGGTGTCTGCAT
RhoA	AGGGAGAAGAACACTTCCGC	GGGCATCTTGTGTTTCCACC
ROCK1	AGGACCAATTCCCGGAGGTA	AGCCAACTCTACCTGCTTTCC
ROCK2	ATCAAACGATATGGCTGGAAG	CCATAGACGGATTGGATTGTTCC
MMP2	AGGACATCAGCGGTAAGACC	GGTAGAGGTAGACCAGCGGA
MMP9	TCGACGTGAAGACGCAGAAG	ACCTGATTCACCTCGTTCCG
TIMP1	GATCTATGCTGCTGGCTGTGA	GTCTGTCCACAAGCAGTGAGT
TIMP2	TTGCAATGCAGACGTAGTGA	GCCTTTCCTGCGATGAGGT
TIMP3	ACGCCTTCTGCAACTCTGAC	AGCCTCGGTACATCTTCATCT
Col1	AGACATCCCACCAGTCACCT	TCACGTCATCGCACAACACA
Col2	CTTGAGACTCAGCCACCCAG	CCGAATGCAGGTTTCACCAG
Col3	AATCATGCCCTACTGGTGGC	CGGGTCCAACTTCACCCTTA

GAPDH, glyceraldehyde‐3‐phosphate dehydrogenase; α‐SMA, α‐smooth muscle actin; β‐MHC, β‐myosin heavy chain; ANP, atrial natriuretic peptide; BNP, brain natriuretic peptide; SERCA2a, sarcoplasmic/endoplasmic reticulum Ca(2+)ATPase 2a; PLN, phospholamban; BCL2, B‐cell lymphoma 2; BCLXL, B‐cell lymphoma‐extra large; Casp3, caspase 3; eNOS, endothelial nitric oxide synthase; VEGFA, vascular endothelial growth factor‐A; HIF1α, hypoxia inducible factor 1α; HIF2α, hypoxia inducible factor 2α; IL‐6, interleukin‐6; TNF‐α, tumour necrosis factor α; IFN‐γ, interferon‐γ; TGF‐β1, transforming growth factor β1; BMPR2, bone morphogenetic protein receptor 2; PAI‐1, Plasminogen activator inhibitor‐1; Id‐1, inhibitor of DNA binding; NOX1, NADPH oxidase 1; NOX2, NADPH oxidase 2; NOX4, NADPH oxidase 4; PCNA, Proliferating cell nuclear antigen; RhoA, Ras homologue gene family member A; ROCK1, rho‐associated, coiled‐coil‐containing protein kinase 1; ROCK2, Rho associated coiled‐coil containing protein kinase 2; MMP2, matrix metalloproteinase‐2; MMP9, matrix metalloproteinase‐9; TIMP1, tissue inhibitor of metalloproteinases 1; TIMP2, tissue inhibitor of metalloproteinases 2; TIMP3, tissue inhibitor of metalloproteinases 3; Col1, collagen type 1; Col2, collagen type 2; Col3, collagen type 3.

### Titin isoform composition

Titin isoform protein composition (i.e. presence of the stiff N2B and compliant N2BA isoforms) was analysed as described previously (Sorop *et al*. 2018). In short, snap frozen LV and RV tissues were weighed and pulverized in liquid nitrogen using a mortar and pestle. Cardiac tissue powder was solubilized in 8 mol L^−1^ urea buffer with dithiothreitol and 50% glycerol solution with protease inhibitors (4 × leupeptin, E‐64 and phenylmethanesulphonyl fluoride). Equal dilutions were calculated based on myosin heavy chain content and homogenate samples were loaded on custom‐made 1% agarose gels. Gels were stained with SYPRO Ruby. Samples were measured in triplicate. Only samples with ≦20% degradation were used. Titin isoforms N2B and N2BA were normalized to the total titin amount and the N2B/N2BA ratio was calculated.

### Statistical analysis

SPSS, version 21.0 (IBM Corp., Armonk, NY, USA) was used for the statistical analysis. Statistical analysis was performed with a mixed model ANOVA, with exercise as a repeated measure and CTEPH as a between group comparison, and exercise × CTEPH as an interaction term for the analysis of haemodynamics. ANOVA with CTEPH as a factor was performed for the MRI data, histology and gene expression. Bonferroni *post hoc* testing was performed when appropriate. The correlation coefficient *r* ^2^ was calculated for the relations between two continuous variables. *P* < 0.05 was considered statistically significant. Data are presented in box and whisker plots with the whiskers reflecting minimum to maximum and the median presented as a line.

## Results

### Cardiac hypertrophy and function

**Figure 2:**
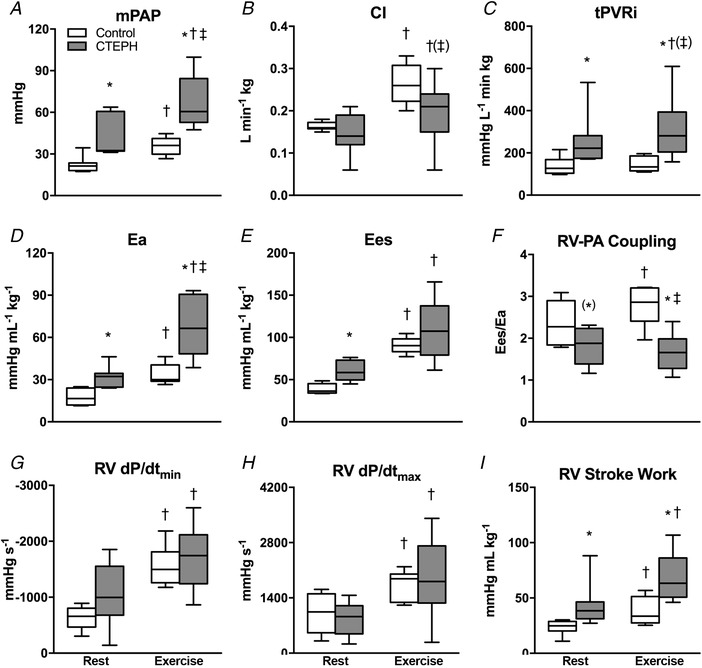
Haemodynamics at rest and during exercise after 9 weeks of CTEPH Data obtained at rest and during maximal exercise at 4 km h^−1^ in control swine (*n* = 7) and CTEPH swine (*n* = 7). *A*, mean pulmonary artery pressure (mPAP). *B*, cardiac index (CI). *C*, total pulmonary vascular resistance index (tPVRi). *D*, arterial elastance (*E*_a_). *E*, end‐systolic elastance (*E*_es_). *F*, RV–PA coupling (*E*_es_/*E*_a_). *G*, maximum rate of fall of RV pressure (RV *dP*/*dt*_min_). *H*, maximum rate of rise of RV pressure (RV *dP*/*dt*_max_). *I*, stroke work at rest and during exercise. Whiskers denote minimum to maximum and the median is indicated by the line. ^*^ *P* < 0.05, ^(*)^ *P* < 0.1 CTEPH *vs*. corresponding control; †*P* < 0.05, (†)*P* < 0.1 Exercise *vs*. corresponding rest; ‡*P* < 0.05, (‡)*P* < 0.1 Exercise × CTEPH (i.e. effect of exercise on variable is different in CTEPH from control).

**Figure 3:**
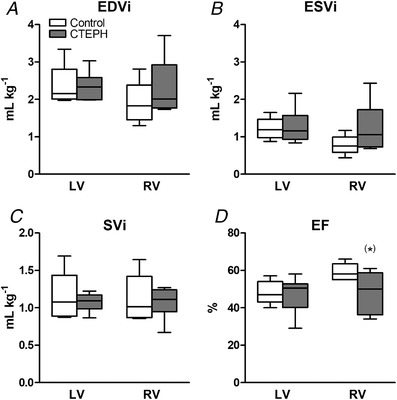
RV and LV dimensions and function measured by CMR imaging *A*, end‐diastolic volume index (EDVi). *B*, end‐systolic volume index (ESVi). *C*, stroke volume index (SVi). *D*, ejection fraction (EF). Whiskers denote minimum to maximum and the median is indicated by the line. Control, *n* = 5; CTEPH, *n* = 6. ^(*)^ *P* < 0.1 CTEPH *vs*. control.

CTEPH resulted in an increased RV afterload, as indicated by an increase in mPAP, tPVRi and *E*_a_ (Fig. 2). This sustained increase in afterload resulted an increase in RV‐BNP expression (Table 2), suggestive of increased RV wall stress. Indeed, trends towards RV dilatation (*P* = 0.15) and decreased EF (*P* = 0.08) as measured with CMR were observed (Fig. 3). However, end‐systolic elastance (*E*_es_), an index of RV contractility, was higher in CTEPH, whereas RV *dP*/*dt*_max_ and RV *dP*/*dt*_min_ were unchanged (Fig. 2). Although RV–PA coupling was reduced, CI was maintained in CTEPH (Fig. 2). Furthermore, heart rate, mAoP (Table 3), LV volume, LVEF (Fig. 3) and LV‐BNP expression (Table 2) were not altered.

**Table 2:** Relative gene expression in control and CTEPH left and right ventricle tissue

		LV					RV				
		control	*n*	CTEPH	*n*	*P* value	control	*n*	CTEPH	*n*	*P* value
Hypertrophy and contractility	α‐SMA	0.36 ± 0.03	9	0.39 ± 0.08	6	0.72	0.17 ± 0.04	9	0.45 ± 0.23	5	0.14
	β‐MHC	1.06 ± 0.08	9	0.94 ± 0.14	6	0.43	1.02 ± 0.07	9	1.10 ± 0.10	5	0.50
	ANP	0.34 ± 0.07	8	0.29 ± 0.07	6	0.65	0.81 ± 0.18	9	0.45 ± 0.14	5	0.20
	BNP	0.11 ± 0.03	9	0.22 ± 0.07	6	0.12	0.12 ± 0.06	9	0.90 ± 0.23	5	***0.001***
	SERCA2a	1.05 ± 0.09	9	1.08 ± 0.12	6	0.88	0.77 ± 0.10	9	0.78 ± 0.25	5	0.97
	PLN	1.40 ± 0.07	9	1.28 ± 0.10	6	0.30	1.32 ± 0.13	9	1.26 ± 0.12	5	0.76
	PLN/SERCA2a	1.39 ± 0.12	9	1.21 ± 0.06	6	0.25	1.87 ± 0.19	9	1.95 ± 0.29	5	0.81
Apoptosis	BCL2	0.57 ± 0.04	9	0.72 ± 0.09	6	*0.10*	0.86 ± 0.06	9	1.16 ± 0.12	5	***0.03***
	BCLXL	0.74 ± 0.08	9	0.62 ± 0.15	6	0.48	1.07 ± 0.10	9	0.87 ± 0.13	5	0.26
	Casp3	0.74 ± 0.06	9	0.70 ± 0.06	6	0.64	0.88 ± 0.05	9	1.22 ± 0.16	5	***0.03***
Endothelial function and angiogenesis	eNOS	0.94 ± 0.06	9	0.87 ± 0.13	6	0.61	0.77 ± 0.05	9	0.81 ± 0.06	5	0.56
	VEGFA	1.16 ± 0.12	9	1.36 ± 0.14	6	0.31	0.93 ± 0.09	9	1.25 ± 0.14	5	*0.07*
	HIF1α	1.19 ± 0.03	9	1.14 ± 0.12	6	0.61	1.14 ± 0.04	9	1.06 ± 0.05	5	0.20
	HIF2α	1.00 ± 0.02	9	0.91 ± 0.11	6	0.35	1.20 ± 0.10	9	1.16 ± 0.12	5	0.81
Inflammation	IL‐6	0.24 ± 0.06	9	0.90 ± 0.57	6	0.19	0.09 ± 0.04	9	0.31 ± 0.25	5	0.26
	TNF‐α	0.22 ± 0.06	9	0.31 ± 0.11	6	0.46	0.27 ± 0.04	9	0.35 ± 0.12	5	0.40
	IFN‐γ	0.84 ± 0.20	9	0.48 ± 0.13	6	0.20	0.44 ± 0.09	9	0.31 ± 0.06	5	0.33
TGF‐β and BMP	TGF‐β1	0.80 ± 0.05	9	0.79 ± 0.04	6	0.92	0.81 ± 0.02	9	0.91 ± 0.06	5	*0.08*
	BMPR2	0.92 ± 0.09	9	0.79 ± 0.09	6	0.35	1.04 ± 0.05	9	1.15 ± 0.06	5	0.22
	PAI‐1	0.38 ± 0.05	9	0.46 ± 0.09	6	0.42	0.23 ± 0.08	9	0.48 ± 0.14	5	*0.10*
	Id‐1	0.49 ± 0.05	9	0.47 ± 0.12	6	0.92	0.61 ± 0.09	9	0.75 ± 0.20	5	0.50
Oxidative stress	NOX1	1.11 ± 0.26	9	1.33 ± 0.42	6	0.64	0.71 ± 0.13	9	1.20 ± 0.17	5	***0.04***
	NOX2	0.45 ± 0.04	9	0.61 ± 0.09	6	*0.08*	0.63 ± 0.09	9	0.68 ± 0.11	5	0.70
	NOX4	0.97 ± 0.08	9	1.13 ± 0.09	6	0.24	0.70 ± 0.06	9	1.03 ± 0.15	5	***0.03***
	PCNA	0.95 ± 0.07	9	0.82 ± 0.07	6	0.25	1.04 ± 0.09	9	1.10 ± 0.22	5	0.79
	RhoA	1.13 ± 0.22	9	1.13 ± 0.03	6	0.72	1.12 ± 0.04	9	1.05 ± 0.06	5	0.36
	ROCK1	1.23 ± 0.05	9	1.22 ± 0.04	6	0.91	1.06 ± 0.06	9	1.11 ± 0.11	5	0.62
	ROCK2	0.76 ± 0.05	9	0.93 ± 0.06	6	*0.05*	0.61 ± 0.04	9	0.95 ± 0.11	5	***0.004***
Extracellular matrix and fibrosis	MMP2	1.30 ± 0.18	6	1.65 ± 0.49	4	0.46	0.89 ± 0.13	9	0.72 ± 0.05	5	0.38
	MMP9	0.89 ± 0.23	9	1.27 ± 0.49	6	0.45	0.61 ± 0.16	8	0.58 ± 0.06	4	0.89
	TIMP1	0.65 ± 0.11	9	0.65 ± 0.11	6	0.75	0.69 ± 0.14	9	0.78 ± 0.13	5	0.69
	TIMP1/MMP9	1.13 ± 0.25	9	0.90 ± 0.28	6	0.56	1.79 ± 0.40	8	1.49 ± 0.43	4	0.65
	TIMP2	1.03 ± 0.10	9	1.24 ± 0.22	6	0.35	0.89 ± 0.12	9	0.95 ± 0.08	5	0.74
	TIMP2/MMP2	0.90 ± 0.12	6	0.84 ± 0.05	4	0.69	1.04 ± 0.05	9	1.35 ± 0.12	5	***0.02***
	TIMP3	1.27 ± 0.08	9	1.08 ± 0.06	6	0.12	0.79 ± 0.05	9	0.76 ± 0.08	5	0.75
	Col1	0.94 ± 0.14	9	1.11 ± 0.50	6	0.70	0.56 ± 0.16	9	0.44 ± 0.17	5	0.63
	Col2	0.85 ± 0.15	9	1.12 ± 0.49	6	0.54	0.65 ± 0.15	9	0.46 ± 0.15	5	0.42
	Col3	0.95 ± 0.14	9	1.13 ± 0.50	6	0.70	0.73 ± 0.16	9	0.43 ± 0.12	5	0.24
	Col1/3	0.99 ± 0.05	9	0.98 ± 0.03	6	0.88	0.75 ± 0.06	9	0.95 ± 0.09	5	*0.09*

Relative gene expression of right and left ventricle tissue obtained from control and CTEPH swine. For abbreviations, see Table 1. Data are mean ± SEM. Bold italic values indicate *P* < 0.05, italic values indicate *P* < 0.10

**Table 3:** Systemic haemodynamics at rest and during exercise

		Control	CTEPH
BW	Instrumentation	20 ± 1	23 ± 1
	End of study	61 ± 2	62 ± 3
HR	Rest	136 ± 21	131 ± 4
	Exercise	235 ± 15†	211 ± 8†‡
SVi	Rest	1.28 ± 0.09	1.17 ± 0.17
	Exercise	1.07 ± 0.09†	0.99 ± 0.13
mAoP	Rest	91 ± 4	100 ± 4
	Exercise	96 ± 4(†)	116 ± 4*†(‡)
SVRi	Rest	561 ± 24	839 ± 205
	Exercise	384 ± 25†	752 ± 252

Body weight (BW) and systemic haemodynamic data at rest and during exercise. HR, heart rate; SVi, stroke volume index; mAoP, mean aorta pressure; SVRi, systemic vascular resistance index. Data are mean ± SEM. Control *n* = 7, CTEPH *n* = 7. ^*^ *P* < 0.05 CTEPH *vs*. corresponding control; †*P* < 0.05, (†)*P* < 0.1 Exercise *vs*. corresponding rest; ‡*P* < 0.05, (‡)*P* < 0.1 Exercise × CTEPH (i.e. effect of exercise on variable is different in CTEPH from control).

**Figure 4:**
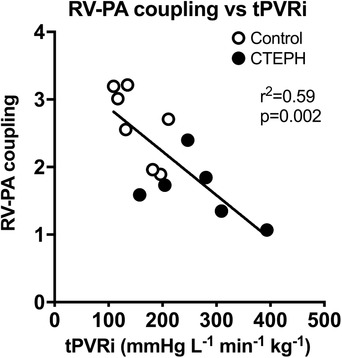
Correlation between total pulmonary vascular resistance (tPVRi) and RV–PA coupling Correlation between total pulmonary vascular resistance (tPVRi) and RV–PA coupling during maximal exercise at 4 km h^−1^ in control swine (*n* = 7) and CTEPH swine (*n* = 6). The *P* value denotes the significance of the slope from zero.

Exercise resulted in increases in mPAP and Ea that were larger in CTEPH compared to control, whereas the exercise induced increase in CI was blunted. Moreover, although *E*_es_ increased in both CTEPH and control animals, *E*_es_ was no longer different between groups. Hence, RV–PA coupling, which increased with exercise in the control swine, did not change significantly during exercise in CTEPH animals (Fig. 2). Indeed, RV–PA coupling worsened with exercise in four out of six CTEPH animals, and correlated inversely with tPVRi (Fig. 4). Moreover, the CTEPH animal with the worst RV function was uncapable of performing exercise at 4 km h^−1^ as a result of RV failure, as indicated by a significant reduction in mAoP during exercise (animal not included in Fig. 4). Altogether, these data are consistent with RV dysfunction that is still compensated for at rest but is excacerbated during exercise.

**Figure 5:**
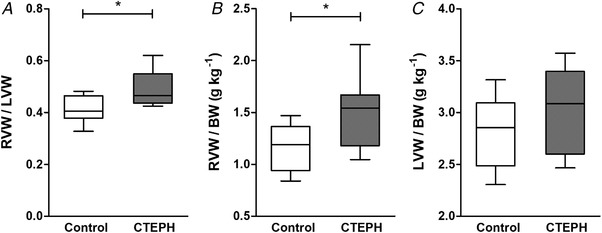
Cardiac hypertrophy (A) Fulton index calculated as the ratio of right ventricular weight (RVW) and left ventricular weight (LVW) and (*B*) RVW over body weight (BW) were increased at death in CTEPH swine, whereas (*C*) LVW over BW was similar in CTEPH and control swine. Whiskers denote minimum to maximum and the median is indicated by the line. Control, *n* = 9; CTEPH, *n* = 8. ^*^ *P* < 0.05 CTEPH *vs*. control.

**Figure 6:**
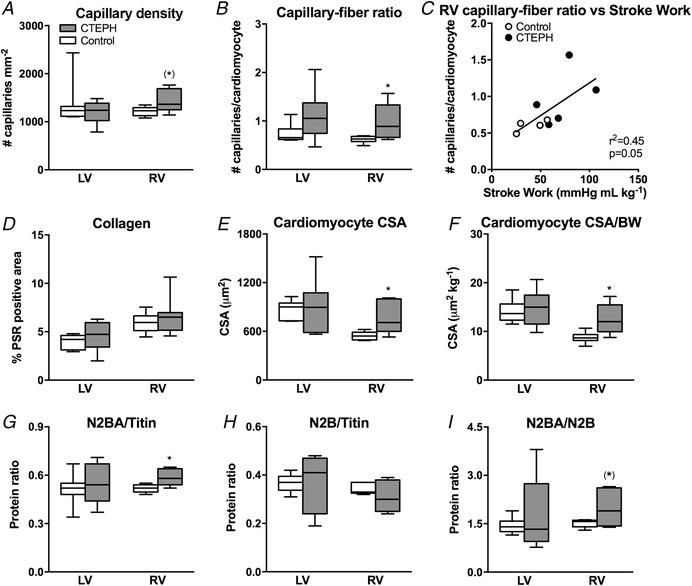
Histological analyses in control and CTEPH animals of both the left ventricle (LV) and right ventricle (RV) *A*, capillary density per mm^2^ (lectin staining). *B*, capillary–fiber ratio. *C*, correlation between stroke work during maximal exercise at 4 km h^−1^and RV capillary–fiber ratio. *D*, interstitial fibrosis [picrosirius red (PSR) staining]. *E*, cardiomyocyte size. *F*, cardiomyocyte size normalized for body weight (Gomori staining, CSA). Myofilament composition in terms of the two different titin isoforms. *G*, N2BA (N2BA/Titin). *H*, N2B (N2B/Titin). *I*, ratio of N2BA and N2B. Whiskers denote minimum to maximum and the median is presented by the line. Control, *n* = 8; CTEPH, *n* = 6. ^*^ *P* < 0.05 CTEPH *vs*. control ^(*)^ *P* < 0.1 CTEPH *vs*. control.

**Figure 7:**
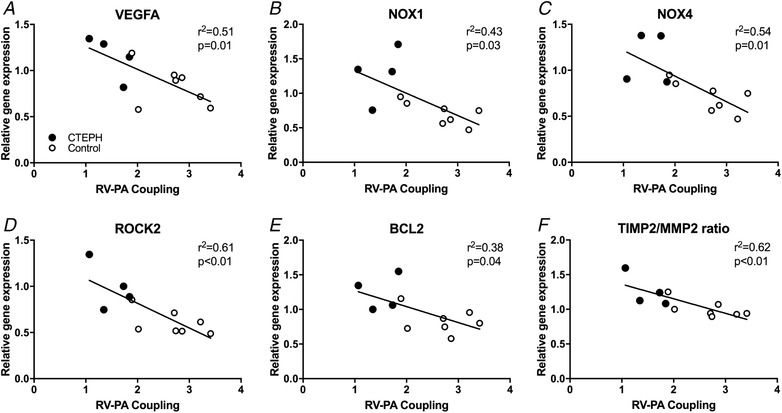
Correlation of the RV–PA coupling during exercise Correlation of the RV–PA coupling during exercise with expression of (*A*) vascular endothelial growth factor A (VEGFA), (*B*) NADPH oxidase 1 (NOX1), (*C*) NADPH oxidase 4 (NOX4), (*D*) Rho‐associated protein kinase 2 (ROCK2), (*E*) B‐cell lymphoma 2 (BCL2) and (*F*) ratio of tissue inhibitor of metalloproteinases 2 (TIMP2) over matrix metalloproteinase‐2 (MMP2) in the RV. Control, *n* = 7; CTEPH, *n* = 4. The *P* value denotes significance of the slope from zero.

As reported previously (Stam *et al*. 2018a), the increased RV afterload resulted in RV hypertrophy, as indicated by an increased RV/BW and Fulton index (Fig. 5), as well as an increased RV cardiomyocyte CSA in CTEPH (Fig. 6). RV cardiomyocyte CSA of CTEPH animals resembled those of LV cardiomyocytes. LV cardiomyocytes were similar in size in LV of CTEPH compared to control animals (Fig. 6), consistent with the maintained LVW/BW in CTEPH compared to control swine (Fig. 5). Expression of SERCA2a, its inhibitor phospholamban (PLN) and their ratio did not change in the RV (Table 2). However, there was a shift in RV titin isoform expression from the stiff N2B to the more compliant N2BA isoform (Fig. 6). The pro‐apoptotic gene caspase‐3 was up‐regulated in the RV, whereas the anti‐apoptotic gene BCL2 was also up‐regulated in the RV of CTEPH animals (Table 2). Expression of BCL2 correlated modestly and inversely with RV–PA coupling during exercise (Fig. 7) but not with resting RV–PA coupling (*r* ^2^ = 0.08; not shown). In the LV, none of the genes involved in cardiac hypertrophy and apoptosis were significantly affected in CTEPH animals.

### Angiogenesis

We observed an increase in capillary density in the RV of swine with CTEPH compared to control (Fig. 6), which correlated with the increased stroke work (Fig. 6) and was consistent with the trend towards increased VEGFA expression (Table 2). Moreover, VEGFA expression correlated inversely with RV–PA coupling during exercise (Fig. 7) but not with resting RV–PA coupling (*r* ^2^ = 0.34; not shown). By contrast, no changes in capillary density or VEGFA expression were observed in the LV.

### Inflammation, oxidative stress and interstitial fibrosis

Although expression of the immunomodulatory genes TNF‐α, IL‐6 and IFN‐γ was not altered in the RV, TGF‐β1 gene expression tended to be higher in the RV of CTEPH swine, whereas BMPRII was not altered (Table 2). Consistent with a perturbation in the TGF‐β–BMP balance, PAI also tended to be increased, whereas Id‐1 did not change (Table 2). This shift in the TGF‐β–BMP balance was accompanied by an increased expression of ROCK2, NOX‐1 and NOX‐4 in the RV (Table 2), indicative of an increase in oxidative stress. Expression of ROCK2, NOX‐1 and NOX‐4 correlated inversely with RV–PA coupling during exercise (Fig. 7) but not with resting RV–PA coupling (*r* ^2 = ^0.29, 0.01 and 0.16 for ROCK2, NOX1 and NOX4, respectively).

These changes in gene expression of pro‐inflammatory genes and genes promoting oxidative stress did not result in overt changes in interstitial fibrosis because collagen content was similar in the RV of CTEPH *vs*. control swine (Fig. 6). Although no change in interstitial fibrosis was observed, there was a trend towards a shift in expression of Col3 to the stiffer Col1 isoform, which was accompanied by an increase in the ratio of TIMP2/MMP2 (Table 2) that correlated inversely with RV–PA coupling (Fig. 7), suggesting reduced ECM turnover in the diseased RV.

With the exception of an increase in ROCK2 and a trend towards an increase in NOX2 expression, no changes in genes involved in inflammation, oxidative stress and fibrosis were observed in the LV (Table 2), which is consistent with the absence of changes in LV myocardial interstitial collagen content.

## Discussion

The present study investigated functional, histological and molecular changes in the RV and LV in swine with CTEPH. The main findings were that CTEPH resulted in (i) RV hypertrophy, both at the global and the myocyte level; (ii) mild RV dysfunction, as indicated by decreased RV–PA coupling and elevated BNP expression, with trends towards an increased RV EDVi and a lower EF; (iii) a further decrease in RV–PA coupling during exercise that correlated with an increase in ROCK2, NOX1 and NOX4 expression; and (iv) increased VEGFA expression that was accompanied by an increased capillary density in the RV. Finally, CTEPH did not result in changes in LV structure or function and was associated with minor changes in LV gene expression in our swine model.

### Animal model

CTEPH was induced in juvenile swine by first inducing endothelial dysfunction through chronic NOS‐inhibition, followed by up to five repeated embolizations with microspheres. We previously showed that this combination was required because neither NOS‐inhibition, nor embolization alone were sufficient to induce chronically elevated pulmonary artery pressures, whereas the combination of NOS‐inhibition and embolization resulted in a progressive increase in tPVRi that continued to increase after the last embolization and was accompanied by pulmonary microvascular remodelling (Stam *et al*. 2018a,*b*). The required induction of endothelial dysfunction may be the result of the younger age of our animals because endothelial NO‐production decreases with age (Liu *et al*. 1992; Parker *et al*. 2000). Also in humans, endothelial dysfunction is often present both in patients with acute pulmonary embolism, as well as with CTEPH, and correlates with disease severity (Reesink *et al*. 2006; In *et al*. 2016; Chibana *et al*. 2017). In humans, CTEPH prevalence is higher in females (Kirson *et al*. 2011), although male patients typically have a worse prognosis (Chen *et al*. 2018). In the present study, male and female swine were used because we have previously shown that there are subtle differences in regulation of pulmonary vascular tone (de Beer *et al*. 2010; de Wijs‐Meijler *et al*. 2017) and hence it is possible that sex also affects development of CTEPH and subsequent RV remodelling in our animals. Unfortunately, the small group size precludes statistical assessment of the effect of sex.

### RV function and remodelling

RV afterload increases during development and progression of pulmonary hypertension. To cope with the increased afterload, the RV undergoes structural and functional changes to augment contractility, and there is evidence that this RV structural and functional adaptability are important determinants of functional capacity and survival in patients with CTEPH (Hardziyenka *et al*. 2011; van de Veerdonk *et al*. 2016; Claeys *et al*. 2019). The effects of CTEPH on cardiac structure, function and gene expression were therefore examined in our porcine model. CTEPH resulted in an increase in RV cardiomyocyte size and global RV hypertrophy, which was accompanied by activation of both pro‐ and anti‐apoptotic gene expression (increases in caspase‐3 and BCL2, respectively). Although these data suggest that apoptosis is probably altered in the remodeled RV, apoptosis is determined by enzyme activity rather than expression. Future experiments examining activity of enzymes involved in apoptosis and TUNEL (terminal deoxynucleotidyl transferase dUTP nick end labelling) staining should be performed to clarify whether the increased mRNA expression is indeed translated to alterations in apoptosis.

Consistent with our previous study in which RV dimensions were assessed using echocardiography in awake swine (Stam *et al*. 2018a), CTEPH resulted in trends towards RV dilatation and a reduced RVEF. In the present study, RV resting function was still preserved, as indicated by a maintained CI, although BNP expression was increased, suggestive of an increased wall stress (Torbicki & Fijałkowska, 2007). These findings are consistent with observations in another porcine CTEPH model, in which CTEPH is induced by ligation of the left pulmonary artery, in combination with embolization of the proximal segmental arteries with glue (Guihaire *et al*. 2013, 2014, 2015). In that model, RV dilatation (Guihaire *et al*. 2015) and RV myocyte hypertrophy (Guihaire *et al*. 2014) were also accompanied by an increased BNP expression (Guihaire *et al*. 2014, 2015), which correlated inversely with stroke volume and positively with global RV hypertrophy (Guihaire *et al*. 2014). Furthermore, RV–PA coupling, an index of how well the RV can cope with the increased afterload, was reduced in that study and a correlation was found between reduced coupling and a reduced SV reserve with dobutamine (Guihaire *et al*. 2015). Similarly, in the present study, severity of CTEPH, as reflected in the tPVRi, correlated inversely with RV–PA coupling during exercise. Importantly, recent studies in patients with CTEPH show that RV–PA coupling correlates with exercise capacity (Claeys *et al*. 2019), which in turn is a strong prognosticator (Blumberg *et al*. 2013).

It is increasingly recognized that not only RV systolic function, but also RV diastolic function correlates with prognosis in patients with pulmonary arterial hypertension (PAH) (Trip *et al*. 2015). Indeed, in pigs with type II pulmonary hypertension (PH), abnormalities in RV–PA coupling were accompanied by diastolic dysfunction (Aguero *et al*. 2014). Diastolic RV chamber stiffness is determined by myocyte stiffness, as well as interstitial collagen. In a rat model of pulmonary artery banding, mild RV dysfunction was accompanied by an increase in myocyte stiffness, whereas interstitial fibrosis was only observed in the presence of severe RV dysfunction (Rain *et al*. 2016). In these rats, the increased myocyte stiffness was accompanied by a paradoxical increase in the more compliant titin N2BA isoform, possibly to blunt a further increase in myocyte stiffness. Consistent with these findings, the mild RV dysfunction in our swine with CTEPH was accompanied by an increase in titin N2BA, whereas no changes in myocardial collagen content were observed histologically. Furthermore, no changes in Col1 and Col3 expression were observed, although there was a change in the ratio between Col1 and Col3 indicating a relatively higher expression of the stiff Col1 isoform. These data are also consistent with the isoform shift observed by Rain *et al*. (2016) and may have contributed to a stiffer RV.

The transition from RV dysfunction to overt RV failure is associated with inflammation and activation of the immune response (Frangogiannis, 2017; Sun *et al*. 2017; Dewachter & Dewachter, 2018). Although expression of genes involved in immune modulation (TNF‐α, IL‐6, IFN‐γ) was not altered, expression of TGF‐β1 tended to be increased. Activation of the TGF‐β pathway was further confirmed by the increase in expression of its downstream target PAI‐1. Both activation of the TGF‐β pathway and increased circulating levels of endothelin, as previously shown to be present in our porcine CTEPH model (Stam *et al*. 2018b), can result in activation of the Rho‐kinase pathway (Zeidan *et al*. 2014; Shimizu & Liao, 2016; Tsai *et al*. 2017). Indeed, ROCK2 expression was up‐regulated in CTEPH swine and showed a strong inverse correlation with RV–PA coupling. ROCK2 activation is involved in cardiac hypertrophy and oxidative stress and also plays a deleterious role in RV remodelling (Ikeda *et al*. 2014; Sunamura *et al*. 2018). ROCK2 phosphorylates protein phosphatase 1, which regulates both myofilament sensitivity to Ca^2+^, as well as Ca^2+^‐handling (Hartmann *et al*. 2015). Hence, although SERCA2a and phospholamban gene expression were not changed in the present study, it is possible that post‐translational modifications in their phosphorylation status contributed to altered Ca^2+^ handling. Indeed, it has been suggested that changes in Ca^2+^‐handling may play a role in the development of RV dysfunction because diastolic dysfunction in swine with type II PH was associated with reduced SERCA2a‐expression (Aguero *et al*. 2014). Additional studies in our CTEPH model are required to further investigate the post‐translational modifications in contractile and Ca^2+^‐handling proteins.

Another key factor that distinguishes adaptive RV remodelling from RV failure is myocardial angiogenesis (Frump *et al*. 2018). Angiogenesis allows RV perfusion to be enhanced commensurate with the increase in RV mass. Indeed, many studies have shown that RV failure is accompanied by a reduction in capillary density, whereas capillary density is preserved or even increased in adaptive RV remodelling (an overview of angiogenesis in the RV in a variety of animal models with PH is provided by Frump *et al*. 2018). Although chronic administration of l‐NAME could significantly reduce myocardial angiogenesis (Matsunaga *et al*. 2002) and limit myocardial perfusion, capillary density was actually increased in the RV of CTEPH swine and correlated with stroke work during exercise. These data are in accordance with recent data obtained in another porcine CTEPH model (Loisel *et al*. 2019) suggesting a state of adaptive RV remodelling with sufficient myocardial perfusion and oxygenation under resting conditions. Nevertheless, VEGFA‐expression was higher in swine with CTEPH (Loisel *et al*. 2019; present study) and correlated with RV–PA coupling during exercise, suggesting that, even though expression of HIF‐1α and HIF‐2α was unchanged, there was still a need for additional perfusion during stress. Indeed, myocardial perfusion reserve has been shown to be reduced in humans with CTEPH and PAH (van Wolferen *et al*. 2008; Vogel‐Claussen *et al*. 2011). Furthermore, myocardial perfusion reserve correlated inversely with mPAP and RV work in these studies, suggesting that flow reserve is recruited as a result of the increased work (Vogel‐Claussen *et al*. 2011) and maximal flow may be limited as a result of increased extravascular compression (van Wolferen *et al*. 2008).

ROCK2 is not only expressed in the myocardium, but also in the vasculature, where its expression correlates with oxidative stress and NOX‐expression (Chen *et al*. 2017). NOX1, NOX2 and NOX4 were up‐regulated in the right coronary artery of swine with pulmonary artery banding, which was accompanied by oxidative stress and endothelial dysfunction, despite maintained eNOS expression (Lu *et al*. 2011). The up‐regulation of NOX1 and NOX4, as well as the unaltered eNOS expression in the RV of CTEPH swine, as observed in the present study, are consistent with these data, although we did not determine the exact intramyocardial location of their expression. Furthermore, up‐regulation of NOX4 is also consistent with recent data from patients with PAH, in which circulating NOX4 was increased (He *et al*. 2017). Finally, the correlation of NOX1 and NOX4 with RV–PA coupling suggests that oxidative stress in the myocardium may contribute to worsening of RV function.

## Conclusion and clinical implications

In swine with CTEPH, the increased afterload resulted in RV hypertrophy, which contributed to a maintained resting RV function, although a trend towards RV dilatation and reduced RVEF was observed with CMR. Consistent with data obtained in CTEPH patients without overt RV failure (Hardziyenka *et al*. 2011), neither LV function, nor LV gene expression (perhaps with exception of ROCK2, NOX2 and BCL2) were altered.

CTEPH is different from PAH in that patients often experience an acute thromboembolic event prior to development of the disease. This form of PH therefore has the potential for follow‐up and earlier therapeutic interventions. Exercise unmasked mild RV dysfunction, as indicated by reduced RV–PA coupling, which may facilitate early diagnosis of patients at risk for developing persistent RV failure. The present study shows that this mild RV dysfunction correlates with changes in expression of genes involved in oxidative stress, apoptosis and angiogenesis. These changes in gene expression suggest activation of an inflammatory response in the RV, promoting oxidative stress. Given that ROCK2 shows a strong correlation with RV dysfunction and has been shown to play a detrimental role in inflammation, oxidative stress, interstitial fibrosis, cardiac hypertrophy and impaired myocardial perfusion, ROCK2 inhibition may provide a viable target for early therapeutic intervention.

## Additional information

### Competing interests

The authors declare that they have no competing interests.

### Author contributions

KS, ZC, JvdV, AH, DJD and DM were responsible for the study design. KS, ZC, NvdV, RvD, EL and DM were responsible for performing the experiments. KS, ZC, NvdV, RvD, EL, JvdV, AH, DJD and DM were responsible for data quality and analysis. KS and ZC were responsible for writing the manuscript. KS, ZC, NvdV, RvD, EL, JvdV, AH, DJD and DM were responsible for manuscript revision. KS, ZC, NvdV, RvD, EL, JvdV, AH, DJD and DM were responsible for final approval of the paper submitted for publication.

### Funding

This work was supported by the Netherlands Cardiovascular Research Initiative, the Dutch Heart Foundation, the Dutch Federation of University Medical Centers, the Netherlands Organization for Health Research and Development, and the Royal Netherlands Academy of Science. CVON (2012‐08), PHAEDRA. This work was further supported by the China Scholarship Council (201606230252).
